# Characterization of crude cellulase produced by *Bacillus licheniformis* OE-1 from the Diyadin hot springs, Ağrı Province

**DOI:** 10.1007/s10123-026-00812-5

**Published:** 2026-04-10

**Authors:** Osman Eren, Halis Sakiroglu

**Affiliations:** 1 Faculty of Science and Literature Department of Chemistry, Ağrı İbrahim Çeçen University, Ağrı, Turkey; 2 Faculty of Health Sciences Department of Nutrition and Dietetics, Ağrı İbrahim Çeçen University, Ağrı, Turkey

**Keywords:** Bacillus licheniformis, Cellulase, Diyadin hot springs, Extreme environments, Enzyme kinetics, Vmax, Km, IC50, Thermostability, Biocatalysts

## Abstract

The exploration of extreme environments offers significant potential for discovering enzymes that may be suitable for demanding industrial conditions. In this study, we characterized the crude cellulase produced by the *Bacillus licheniformis* OE-1 strain, isolated from the thermal springs of Diyadin (Ağrı Province, Turkey). The inherent adaptation of this strain to extreme habitats suggests a potential for enzymatic stability. The enzyme exhibited its optimal activity at 60 °C and pH 9.0, indicating its potential for application in certain high temperature and alkaline based processes. Using carboxymethyl cellulose as a substrate, the enzyme displayed a Vmax of 2.29 U mL⁻¹ and a Km of 0.1691 mg/mL. The effects of various compounds on the enzyme were investigated; IC_50_ values were calculated from dose-response curves, and inhibition types were preliminarily identified using Lineweaver-Burk plots. EDTA, MgSO_4_ and KI were found to be uncompetitive inhibitors, while KCl and SDS exhibited non-competitive inhibition. This research highlights the potential of the Diyadin geothermal ecosystem as a candidate source for biocatalysts that may contribute to industrial sustainability.

## Introduction

Cellulose is the most abundant biopolymer on Earth and a major structural component of plant cell walls. Beyond plants, it is also synthesized by various other organisms, including algae, tunicates, and certain bacteria (Moon et al. 2011). Chemically, cellulose is a homopolysaccharide with the empirical formula (C₆H₁₀O₅)ₙ consisting of repeating units of β-D-glucose linked via β-1,4-glycosidic bonds (Hockenberger 2004).

Alkaline and thermotolerant enzymes possess substantial biotechnological potential in the textile, pulp and paper, feed, and food industries due to their ability to maintain catalytic activity under high-temperature and high-pH conditions. In the textile sector, they contribute to more sustainable processing by reducing the need for harsh chemicals during bio-polishing and stone-washing treatments (Singh et al. 2016). In the pulp and paper industry, these enzymes facilitate the degradation of lignocellulosic structures, thereby improving bleaching efficiency and reducing dependence on chlorine-based chemicals (Nagl et al. 2022). In the feed industry, their capacity to hydrolyze fibrous components enhances nutrient digestibility and improves feed conversion ratios (Ariaeenejad et al. 2024). Within the food industry, the application of thermostable enzymes in processes such as juice clarification, viscosity reduction, and the enhancement of aroma and oil extraction efficiency has been shown to shorten processing times and improve overall product quality (Wang et al. 2023; Abdella and Ibrahim 2024; Stanek-Wandzel et al. 2024; Eren et al. 2025; Guerra et al. 2025). In this context, geothermal water sources represent an important ecological niche for the discovery of microorganisms adapted to high temperatures and extreme environmental conditions, as well as for the identification of thermo-stable enzymes with industrial relevance. Owing to its late geological formation and location within active geothermal belts, Turkey possesses a remarkably rich potential for extremophilic microorganisms and industrially valuable enzymes (Ulucay et al. 2022; Ulucay 2025).

Cellulase, a member of the hydrolase enzyme family, catalyzes the hydrolysis of β-1,4 linkages in cellulose, generating glucose, cellobiose, and cellooligosaccharides as end products. The efficiency of cellulose hydrolysis by cellulase plays a pivotal role in determining product yield and quality across diverse industrial sectors. In the food industry, for instance, cellulase enhances both yield and quality in the production of fruit juices and vegetable oils particularly in the extraction of high-value oils such as olive oil. Studies have shown that enzymatic treatment with cellulase not only reduces viscosity but also significantly increases oil yield and enhances levels of key bioactive compounds such as tocopherols (Yu et al. 2019; Motuk 2020; Huang et al. 2022). Beyond food processing, cellulase finds widespread application in the pharmaceutical, animal feed, paper, textile, and biofuel industries. In animal nutrition, the inclusion of cellulase in feed formulations has been shown to enhance nutrient bioavailability and feed conversion efficiency. In the bioenergy sector, cellulase plays a critical role in the enzymatic hydrolysis of lignocellulosic biomass for bioethanol production. Given its wide range of industrial applications, cellulase is considered a highly valuable biocatalyst. Although current reports indicate that the textile industry represents the largest market for cellulase, projections suggest a significant rise in its utilization within the food industry soon.Hu The objective of this study is to obtain a cellulase enzyme characterized by high yield, strong substrate affinity, and thermal stability traits that confer considerable commercial and biotechnological value (Saddler et al., 1986; Teeri, 1992; Lynd et al. 2002; Hu et al. 2008; Lavanya et al. 2011; Jayasekara and Ratnayake 2019).

While several studies have characterized enzymes from *B. licheniformis*, there is limited information regarding the specific kinetic and inhibition profiles of strains adapted to the unique geothermal conditions of the Diyadin region. This study aims to fill this gap by providing a preliminary assessment of the OE-1 strain, focusing on its potential stability and kinetic behavior in the presence of various industrial inhibitors.

In this study, the cellulase enzyme produced by *Bacillus licheniformis* OE-1, isolated from the Diyadin thermal springs and preserved at Agri Ibrahim Cecen University, was investigated. The research determined the optimal pH and temperature conditions, storage stability at 4 °C, and kinetic parameters (V_max_ and K_m_). Furthermore, IC_50_ values and potential inhibition modes were characterized for various compounds. The objective was to evaluate the enzyme’s stability and substrate affinity under alkaline and high-temperature conditions, providing a preliminary assessment of its potential as a candidate for industrial applications, particularly within the food industry.

## Materials and methods

### Materials

Sephadex G-100, electrophoresis reagents, silver staining chemicals, Glycine-NaOH buffer, Sodium phosphate buffer, dinitrosalicylic acid (DNS), sodium-potassium tartrate, Coomassie Brilliant Blue G-250 reagent, and glucose were purchased from Sigma-Aldrich (Germany).

### Qualitative screening of cellulolytic activity on solid media

The cellulase-producing capacity of the bacteria was preliminarily evaluated on CMC-LB agar using the Congo Red staining method. The cultures were incubated at 55 °C for 24 h, after which the plates were stained with Congo Red and washed to visualize the hydrolysis zones. These preliminary assays confirmed that the bacteria can produce cellulase (Hürtaş Tatlı 2013).

### Ammonium sulphate precipitation of cellulase enzyme

The isolated strain was incubated in LB broth medium at pH 7.0 and 55 °C for 24 h at 100 rpm. Following the incubation, the culture was centrifuged at 10.000 rpm for 15 min at 4 °C to remove the bacterial cells, and the resulting supernatant was transferred to sterile Falcon tubes. Preliminary assays using the DNS method revealed cellulase activity in the supernatant (Aygan et al. 2011). The resulting pellet was discarded, and ammonium sulphate precipitation was applied to the supernatant in a stepwise manner from 10% to 80% saturation (Türkan et al. 2014). However, since no significant precipitation or activity was observed in the resulting fractions, the crude supernatant was utilized as the primary enzyme source for all further analyses.

### Preparation of crude cellulase enzyme

The bacterial isolate was cultivated in Luria-Bertani (LB) broth at 55 °C and pH 7.0 for 24 h under shaking conditions at 100 rpm. To obtain the crude cellulase, the liquid culture was centrifuged at 10.000 rpm for 15 min. Based on the DNS assay results, the enzyme activity was confirmed to be extracellular (present in the supernatant), and the pellet was discarded. This cell-free supernatant was subsequently utilized as the crude enzyme source for all further characterization assays (Aygan et al. 2011).

### Determination and characterization of cellulase enzyme activity

The enzyme’s optimum temperature, optimum pH, pH stability, storage stability at 4 °C, optimum ion concentration, and kinetic parameters (Kₘ and Vₘₐₓ) were determined using the DNS method (Sumner 1921). The method was applied as follows: the enzyme present in the supernatant and carboxymethyl cellulose (CMC) dissolved in a buffer solution were incubated together in an Eppendorf tube at various pH values and temperatures for 10 min. Subsequently, DNS reagent was added, and the temperature was raised to 100 °C to terminate the reaction. The tubes were then placed in an ice bath for 10 min, and absorbance values were measured at 540 nm. Enzyme activity was calculated either relatively or using the slope obtained from a glucose standard curve. In this method, the DNS reagent undergoes a color change from light yellow to dark orange in direct proportion to the amount of glucose present in the medium. This color change can be detected spectrophotometrically. The incubation times mentioned above were determined through preliminary trials. Glycine-NaOH buffer was used at the optimum fixed pH to determine the optimum ionic strength; the obtained results also include the effects of the buffer ions.

### Glucose Standard Curve

To determine enzyme activity, a standard glucose curve was prepared. Glucose solutions of varying concentrations were made from a stock solution, and their absorbance was measured at 540 nm. Each sample was measured in triplicate, and the arithmetic mean of the three values was used. The resulting data were plotted to generate the standard curve (Khadka et al. 2022).

### Determination of Km and Vmax values of cellulase using CMC substrate

The supernatant was utilized as the enzyme source to determine the kinetic parameters. Measurements were performed under optimal conditions. Enzyme activities at varying CMC concentrations were initially calculated by converting the absorbance values (OD_540_) to µmol glucose equivalents using a glucose standard curve (y = 0.1631x). Subsequently, K_m_ and V_max_ values were determined using a Lineweaver-Burk double-reciprocal plot (1/V versus 1/[S]). The V_max_ and K_m_ values were calculated mathematically from the y-intercept (1/Vmax) and from the x-intercept (-1/K_m_) of this linear plot. (Türkan et al. 2014; Khadka et al. 2022).

### Determination of kinetic parameters of cellulase enzyme

Enzyme activity measurements were performed in the temperature range of 12.5–90 °C. The logarithms of the obtained activity values (log K) were calculated, and the temperature was expressed as 1/T × 1000. Using the resulting plot, an Arrhenius curve was constructed, and the activation energy (E_a_) was determined from the slope of this curve. The activation enthalpy (ΔH) was calculated using the equation ΔH = E_a_ - R × T. Subsequently, the Q₁₀ value was determined, which represents the change in enzyme activity over a 10 °C temperature interval. Q₁₀ is calculated by dividing the higher activity value by the lower activity value measured between two temperatures differing by 10 °C. For the Q_10_ value, activity measurements at 20 °C and 30 °C were considered. The slope of the Arrhenius plot is given by the equation:

$$\:Sloope=\frac{-Ea}{2.303*R}$$

Where E_a_ is the activation energy and R is the universal gas constant (R: 1.987 cal·mol⁻¹·K⁻¹) (Wilson 1971; Segel et al., 1975; Danişan et al., 2004; Keha and Kufrevioglu 2009).

### Partial purification of cellulase enzyme by gel filtration chromatography

Gel filtration chromatography was used for the partial purification of the cellulase enzyme from the bacterium isolated from the Diyadin thermal springs. Sephadex G-100 was used as the gel matrix (Türkan et al. 2014).

### Qualitative protein monitoring by the warburg method

The protein content in the eluates obtained after gel filtration chromatography was monitored qualitatively using the Warburg-Christian method (Segel and Woodin 1968). This method was strictly employed for the rapid screening and identification of protein-containing fractions during the purification process. The presence of protein in the samples was monitored by measuring the absorbance at 280 nm, which primarily detects aromatic amino acid residues. These measurements were used as a fast-screening tool to identify protein-rich eluates to be pooled for further analysis, rather than for the precise quantitative determination of specific activity.

### SDS-PAGE polyacrylamide gel electrophoresis

The purification fractions were analyzed by SDS-PAGE using a 12% resolving gel and a 5% stacking gel. Following the electrophoresis, the protein bands were attempted to be visualized using a high-sensitivity silver staining protocol (Balcı, 2019). However, due to the extremely low protein concentration in the purified enzyme fractions, distinct protein bands could not be clearly visualized on the gel. Therefore, the molecular weight of the enzyme could not be precisely determined via SDS-PAGE in this study.

### Quantitative protein determination by the Bradford method

To calculate the fundamental parameters of the enzyme, including total activity, specific activity, total protein, and purification fold, the protein concentration was determined quantitatively using the Bradford method. Absorbance values were measured at 595 nm, and protein content was calculated using a bovine serum albumin (BSA) standard curve. All measurements were performed in triplicate, and the arithmetic mean was used for final calculations (Şentürk et al. 2011).

### Investigation of inhibitory effects of various compounds on cellulase enzyme

The IC_50_ values for EDTA, MgSO_4_, KCl, KI, and SDS were determined using best-fit non-linear regression models (Exponential Decay: y = a.e^− bx^) derived from the dose-response activity data. To ensure the highest statistical accuracy for the observed inhibition profiles, IC_50_ values were calculated by solving the mathematical equations derived directly from the raw dose-response activity data at 50% inhibition, following the methodology described by Türkan (2014). This approach allowed for a precise determination of inhibitory concentrations by accounting for the specific kinetic behavior of the *Bacillus licheniformis* OE-1 cellulase. Furthermore, preliminary K_i_ values were determined to provide a more detailed characterization of the inhibition kinetics. Utilizing the calculated IC_50_ values as a reference to establish appropriate inhibitor concentrations, Lineweaver-Burk (double-reciprocal) plots were constructed. These plots were utilized to identify the specific inhibition type for each compound and to calculate the preliminary K_i_ values, allowing for a more comprehensive assessment of the interaction between these inhibitors and the *Bacillus licheniformis* OE-1 cellulase Türkan et al. (2014).

### Statistical analysis

In the evaluation of the results obtained in this thesis, the SPSS statistical software package (version 23.0.0 for Windows, SPSS Inc., Chicago, Illinois) was used. Differences among group means were analysed using one-way analysis of variance (One-Way ANOVA). The significance of the differences was determined by Duncan’s multiple range test. All experiments were performed in triplicate, and results are expressed as mean ± standard deviation.

## Results

### Cellulase production on solid medium

**Fig. 1:**
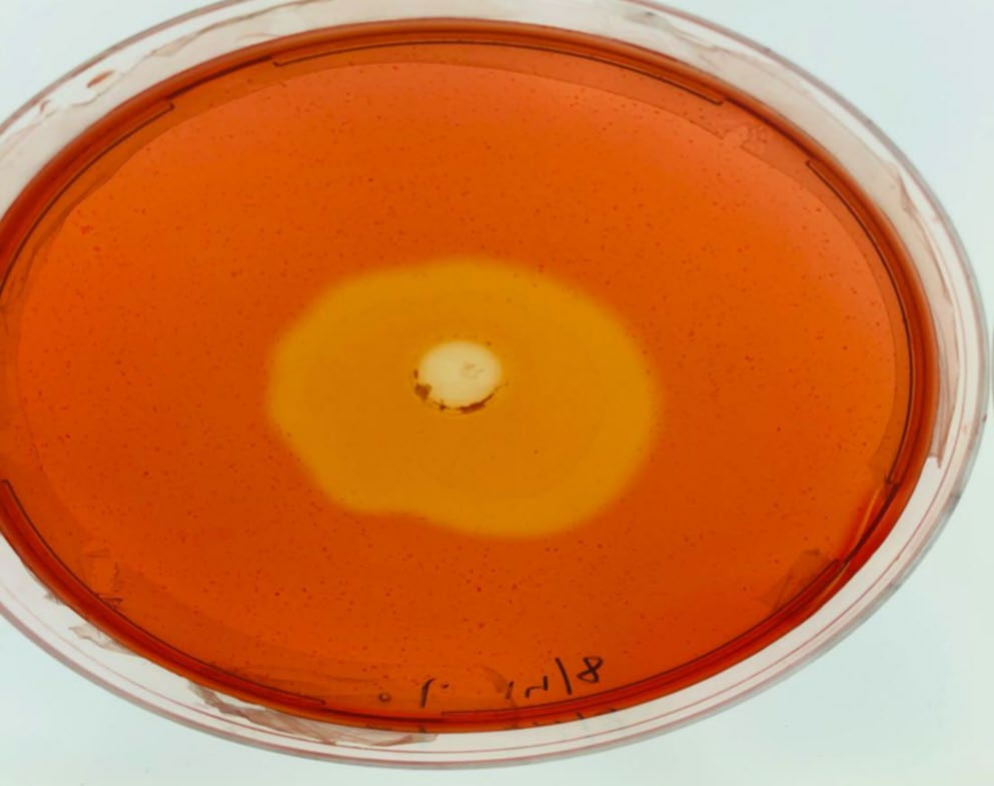
Cellulolytic activity of strain OE on CMC-agar medium. The formation of a distinct clearing zone (diameter: 29 mm) after Congo Red staining resulted in an enzymatic index (EI) of 5.14 (This figure is shared with [ Eren and Sakiroğlu, in press] as it represents the foundational proof of enzyme production for both studies)

The extracellular cellulase activity of the OE-1 strain was assessed on CMC-LB agar plates. Following incubation, Congo Red staining and subsequent NaCl washing revealed distinct hydrolysis zones formed by enzymatic degradation of carboxymethyl cellulose. Clear halos indicating cellulolytic activity were measured to obtain semi quantitative activity values. The OE-1 strain produced a hydrolysis zone of 29 mm with an enzyme index of 5.14, values that were previously reported in our published work (Eren and Sakiroğlu, in press). Literature comparisons indicate that these values are notable. While it is recognized that hydrolysis zones on solid media may not directly reflect liquid culture yields, the obtained results point to a promising enzymatic potential for our strain (Fig. 1).

### Ammonium sulphate precipitation results

Following the cultivation of *B. licheniformis* OE-1 in liquid medium, ammonium sulphate precipitation was applied to the enzyme. The precipitation began with 10% saturation and was increased stepwise up to 80%. However, despite repeating the procedure three times, no enzymatic activity was detected in any of the fractions. This apparent loss of activity was likely due to structural denaturation under high salt concentrations or the interference of residual salts with the DNS reagent used for the activity assay. However, from an industrial perspective, the requirement for extensive and costly purification steps is a significant limiting factor. Rather than pursuing further complex purification, this study focuses on the kinetic and physicochemical characterization of the crude enzyme. The crude cellulase produced by *B. licheniformis* OE-1 could be a potential candidate for direct industrial use due to its stability and kinetic properties. Bypassing expensive purification processes may provide an economic and operational advantage for large-scale applications. Therefore, further experiments were carried out using the crude enzyme present in the supernatant obtained after centrifugation. From this point onward, the term “crude enzyme” refers to this supernatant solution containing the cellulase enzyme.

### Optimum pH of crude cellulase enzyme

**Fig. 2:**
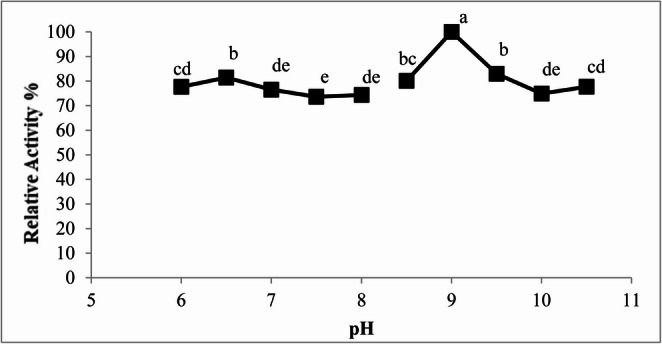
Optimum pH results of the cellulase enzyme (Different letters indicate statistically significant differences according to Duncan’s multiple range test at p < 0.05, n = 3)

The optimum pH was determined by measuring cellulase activity using the DNS method with CMC as the substrate. The results were expressed as relative activity (%), where the pH value yielding the highest activity was defined as 100%. The results indicated that the crude enzyme displays alkaliphilic characteristics, with maximum activity detected at pH 9.0. The significantly increased enzyme activity observed at pH 9.0 may be attributed to favorable electrostatic conditions and optimal protonation states of active-site residues (*p* < 0.05, *n* = 3) (Fig. 2).

### Optimum Ionic Strength Results of the Cellulase Enzyme

**Fig. 3:**
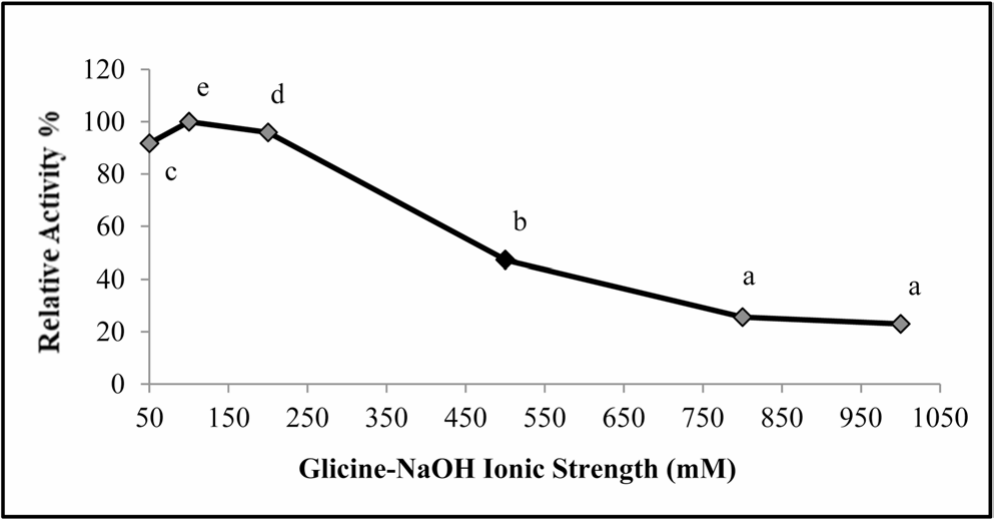
Optimum ionic strength results of cellulase enzyme (Different letters indicate statistically significant differences according to Duncan’s multiple range test at *p* < 0.05, *n*=3)

The effect of ionic strength on the activity of the crude enzyme was determined under optimal pH conditions using the DNS method with CMC as the substrate, and the results were expressed as relative activity (%). Ionic strength analyses were conducted using a Glycine-NaOH buffer system at a fixed pH. The results indicated that the optimum ionic strength for the crude enzyme was 100 mM. While moderate ionic strength levels supported enzyme activity, higher concentrations significantly reduced the activity, likely due to the disruption of electrostatic balance (*p* < 0.05, *n* = 3) (Fig. 3).

### Optimum temperature results of cellulase enzyme

**Fig. 4:**
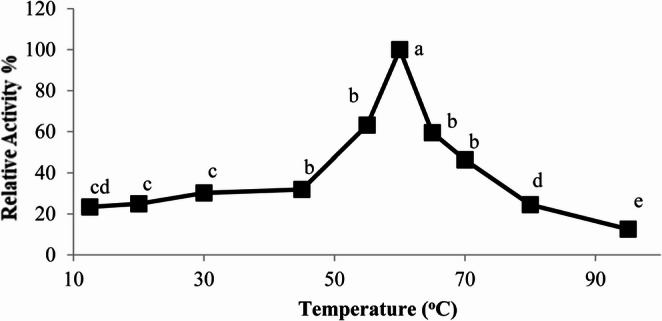
Optimum temperature results of the cellulase enzyme (Different letters indicate statistically significant differences according to Duncan’s multiple range test at *p* < 0.05, *n* = 3.)

Relative activity increased significantly with rising temperature, reaching a maximum at 60 °C. At higher temperatures, a statistically significant decline was observed, and the different letter groupings indicate significant differences among temperatures. (*p* < 0.05, *n* = 3) (Fig. 4).

### Storage stability of the cellulase enzyme at 4 °C

**Fig. 5:**
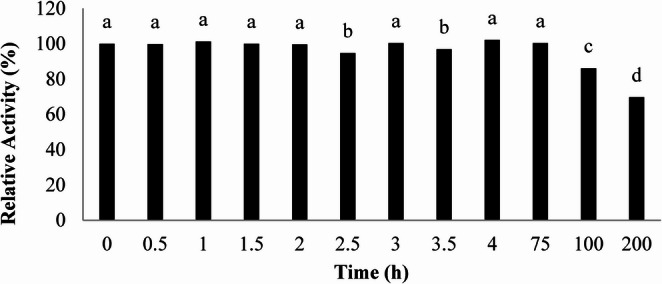
Storage Stability Curve of the Cellulase Enzyme at 4 °C (Different letters indicate statistically significant differences according to Duncan’s multiple range test at *p* < 0.05.)

Enzyme activity was determined using the 3,5-dinitrosalicylic acid (DNS) method by measuring the absorbance of released reducing sugars at 540 nm. The measured activity was evaluated relative to the initial time point (0 h), which was defined as 100%. The sample’s activity remained statistically stable up to 75 h (*p* > 0.05). Although minor yet significant fluctuations were observed at 2.5 and 3.5 h (*p* < 0.05), activity subsequently returned to baseline levels. By 100 h, a significant decline in activity was evident (*p* < 0.05), and at 200 h, the activity had decreased to approximately 70% of its initial value. (*p* < 0.05, *n* = 3) (Fig. 5).

### Glucose standard curve

**Fig. 6:**
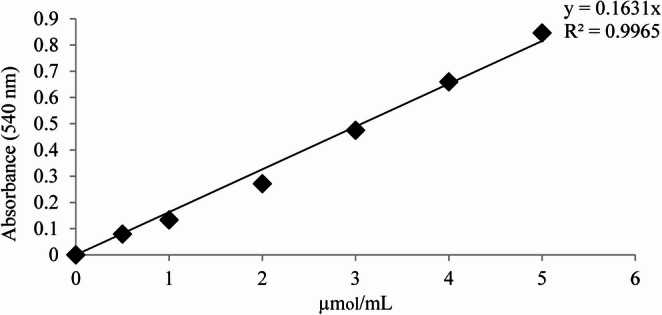
Glucose standard curve

The calculation of enzyme activity requires the slope of the glucose standard curve. For this purpose, a glucose standard curve was prepared by measuring absorbance at 540 nm. (Fig. 6).

#### Results of Km-Vmax, values of cellulase enzyme with CMC substrate

**Fig. 7:**
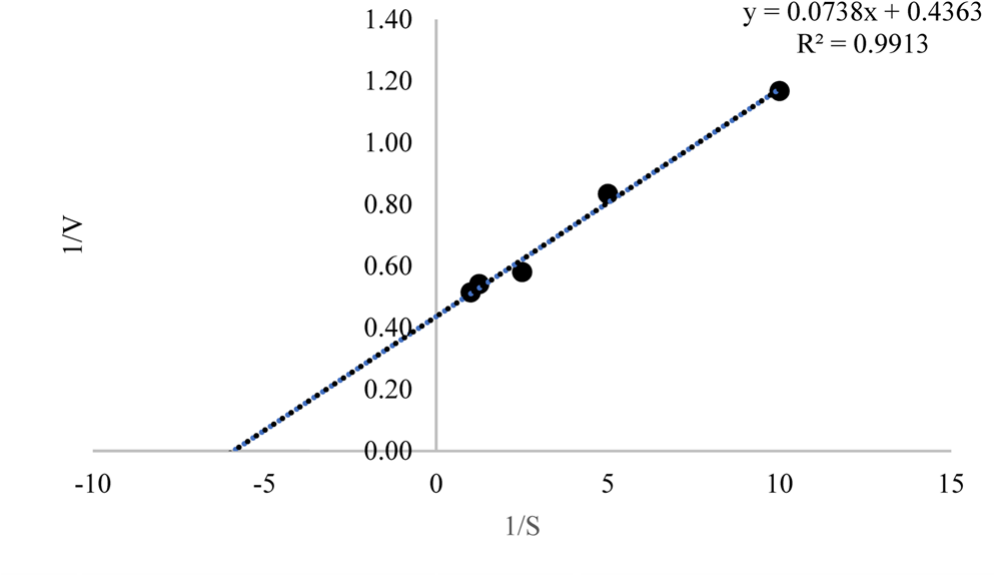
1/V-1/S graph of cellulase enzyme with CMC substrate to determine K_m_-V_max_ values

The supernatant served as the enzyme source and based on the Lineweaver-Burk double-reciprocal plot, the V_max_ of the enzyme was calculated to be 2.29 U mL^− 1^, while the K_m_ was found to be 0.1691 mg/mL (Fig. 7) (Table 1).

**Table 1:** Results of Km and Vmax, values of cellulase enzyme with CMC substrate

Enzyme	Substrate	K_m_ (mg/mL)	V_max_ (U mL⁻¹)
Cellulase	CMC	0.1691	2.29

### Kinetic parameters of the cellulase enzyme

**Fig. 8:**
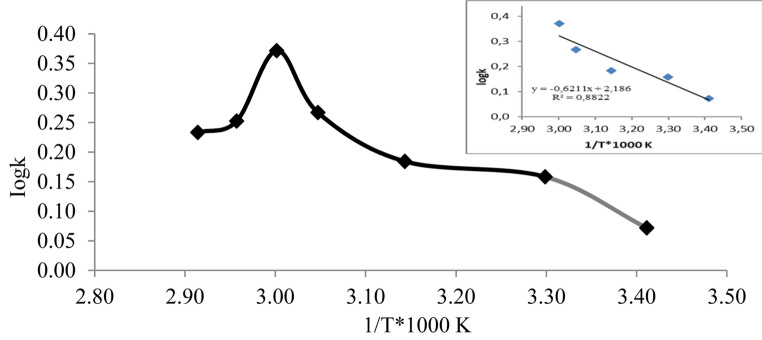
Effect of temperature on cellulase activity and Arrhenius plot (inset) for the determination of activation energy

The activation energy (E_a_), activation enthalpy (ΔH), and Q_10_ values were calculated to determine the thermophysical properties of the crude cellulase. The Ea and ΔH values were found to be 2.82 kcal/mol and 2.16 kcal/mol, respectively. In thermophysical terms, the Ea represents the minimum energy barrier required to initiate substrate hydrolysis, while the ΔH reflects the thermodynamic energy change during the formation of the enzyme-substrate transition state. Additionally, the temperature coefficient (Q_10_), which describes the fold-change in the reaction rate for every 10 °C rise in temperature, was calculated as 1.22 (Fig. 8) (Table 2).

**Table 2:** Optimum temperature, Ea, ∆H, and Q10 values for the cellulose enzyme

Enzyme	Optimum Temperature (°C)	Activation Energy (Ea) (kcal/mol)	Activation Enthalpy (ΔH) (kcal/mol)	Q_10_
Cellulase	60	2.82	2.16	1.22

#### Purification of Cellulase Enzyme Using Sephadex G-100

**Fig. 9:**
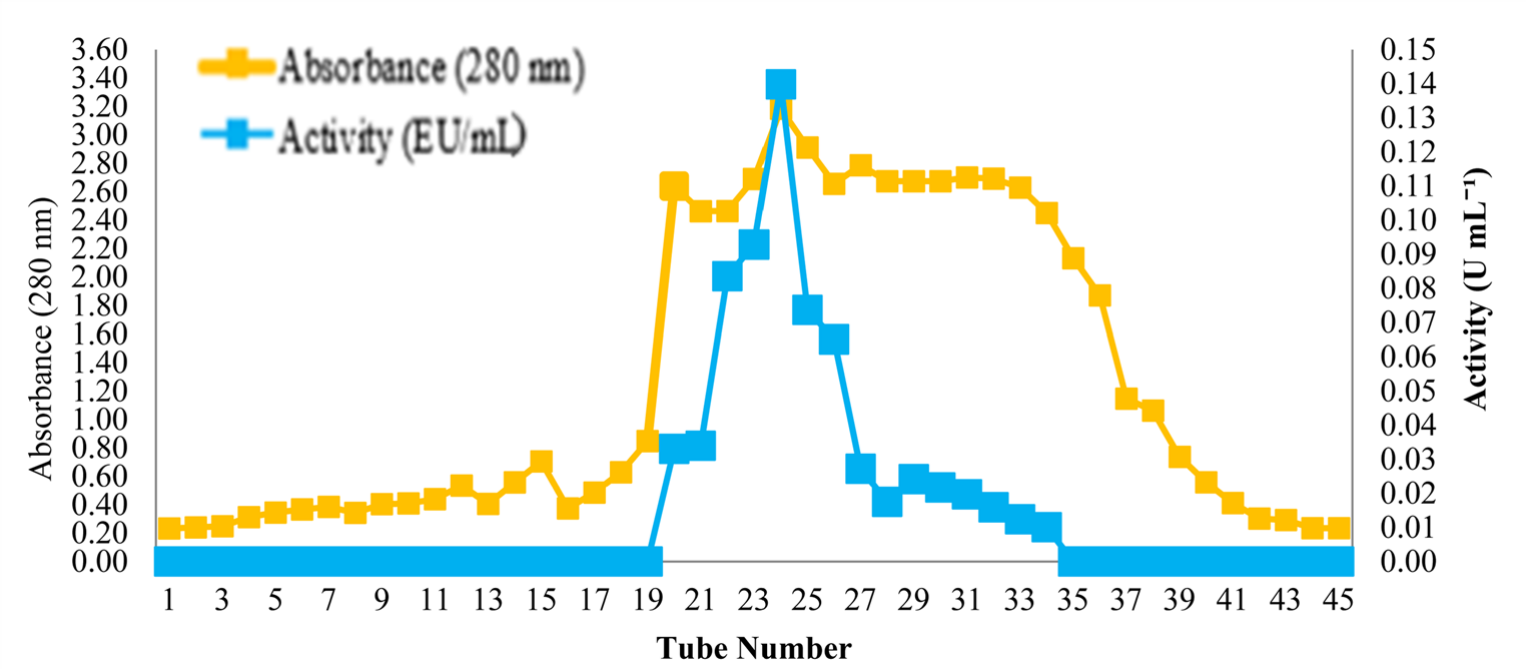
Purification profile of cellulase enzyme obtained by sephadex G-100 gel filtration chromatography

Sephadex G-100 gel filtration chromatography was applied to the crude cellulase enzyme with the aim of increasing Specific Activity. Following the chromatography, parameters including enzyme activity, protein concentration, Specific Activity, and purification fold were calculated. The results showed that the Specific Activity changed from 4.03 to 2.85 U µg^− 1^ protein, with a purification fold of 0.71 (Table 3) (Fig. 9).

### Protein determination using the Bradford assay

**Fig. 10:**
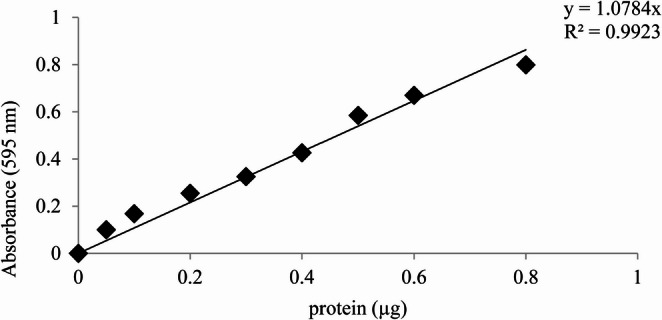
Protein standard curve

Protein concentration was quantified by the Bradford assay using Bovine Serum Albumin (BSA) as the standard to evaluate key parameters including total protein content, specific activity, purification fold, and yield of both crude cellulase and the cellulase fraction obtained after gel filtration chromatography. A standard calibration curve using known concentrations of a reference protein was constructed to facilitate accurate quantification. The results are summarized as follows (Fig. 10) (Table 3).

**Table 3:** Purification table of cellulase enzyme from homogenate and gel filtration results

Sample Type	Total Volume (mL)	Activity (U mL^− 1^)	Protein (µg/mL)	Total Activity (U)	Total Protein (µg)	Specific Activity (U µg^− 1^ protein)	Yield %	Purification Fold
Homogenate	5	2.36	0.58	11.7	2.9	4.03	100	1.00
Gel filtration chromatography	5	0.20	0.07	1	0.3588	2.85	8.54	0.71

### SDS-PAGE analysis of the cellulase enzyme

**Fig. 11:**
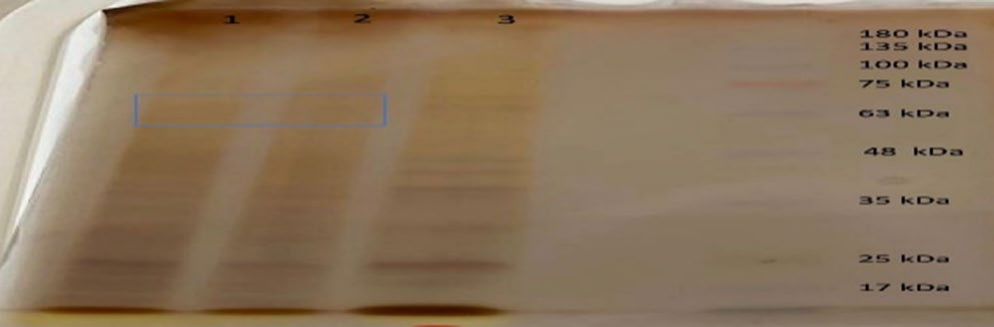
SDS-PAGE gel image of cellulase enzyme (Lanes 1–2: Enzyme samples obtained after gel filtration; Lane 3: Crude enzyme sample from homogenate)

The purity of the cellulase enzyme after gel filtration was evaluated by SDS-PAGE. The analysis revealed multiple faint protein bands rather than a single distinct band. These results indicate that while the total protein content was reduced, the target cellulase was only partially purified and remained at a concentration near the detection limit of the staining method. This visual profile is consistent with the low purification fold and specific activity values reported in the previous section. (Fig. 11).

## IC_50_ values of various compounds on cellulase enzyme activity

**Fig. 12:**
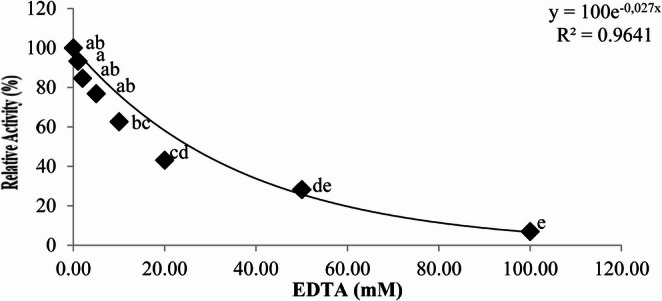
Effect of various EDTA concentrations on the relative activity of cellulase (Different letters indicate statistically significant differences according to Duncan’s multiple range test at *p* < 0.05,*n* = 3)

**Fig. 13:**
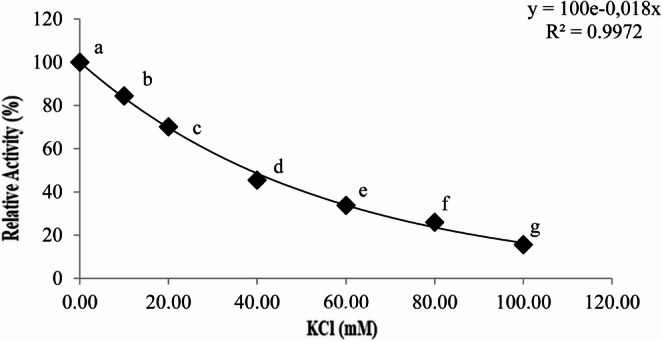
Effect of various KCl concentrations on the relative activity of cellulase (Different letters indicate statistically significant differences according to Duncan’s multiple range test at *p* < 0.05, *n* = 3)

**Fig. 14:**
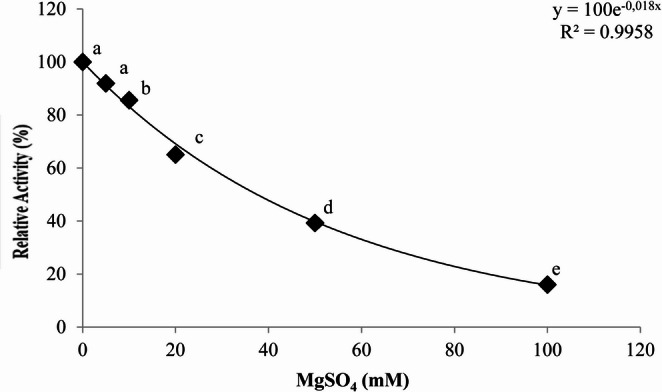
Effect of various MgSO_4_ concentrations on the relative activity of cellulase (Different letters indicate statistically significant differences according to Duncan’s multiple range test at *p* < 0.05, *n* = 3)

**Fig. 15:**
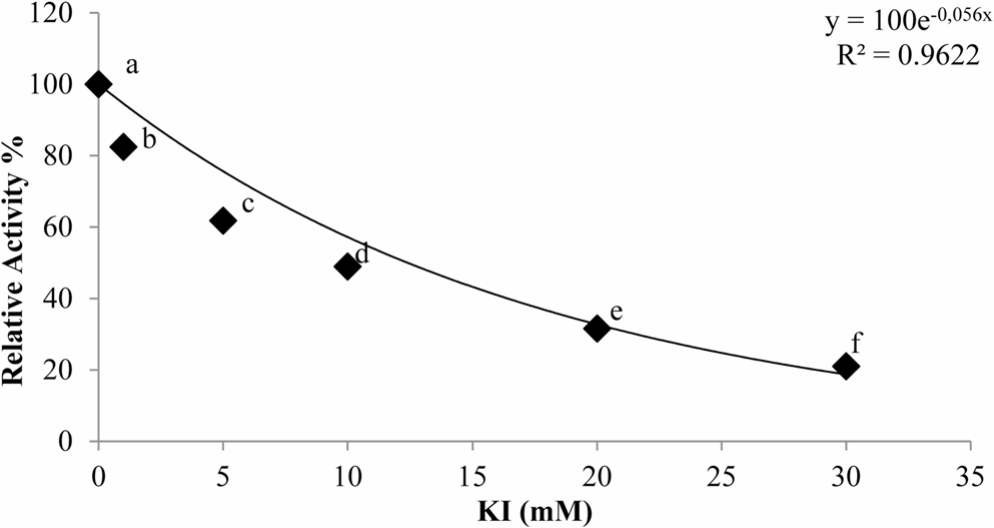
Effect of various KI concentrations on the relative activity of cellulase (Different letters indicate statistically significant differences according to Duncan’s multiple range test at *p* < 0.05, *n* = 3)

**Fig. 16:**
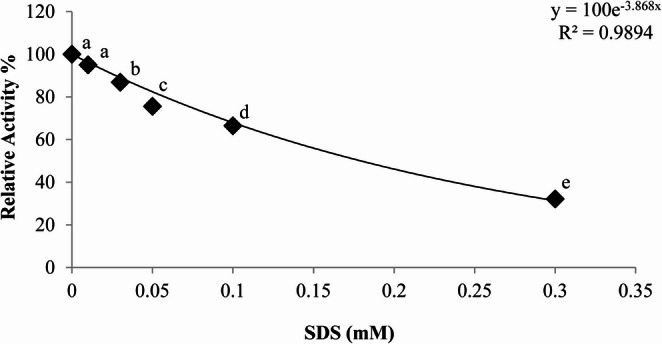
Effect of various SDS concentrations on the relative activity of cellulase (Different letters indicate statistically significant differences according to Duncan’s multiple range test at *p* < 0.05, *n* = 3)

**Fig. 17:**
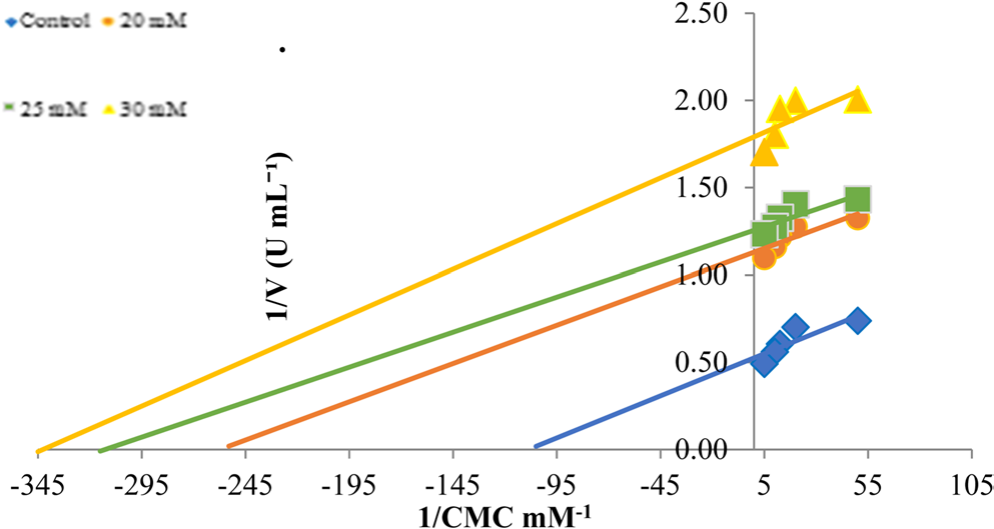
Inhibitory effect of EDTA on cellulase enzyme

**Fig. 18:**
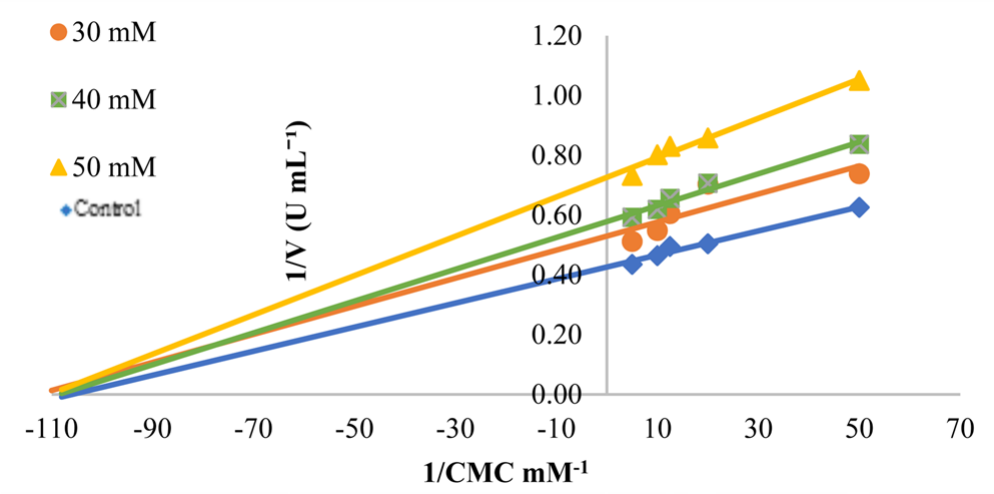
Inhibitory effect of KCl on cellulase enzyme

**Fig. 19:**
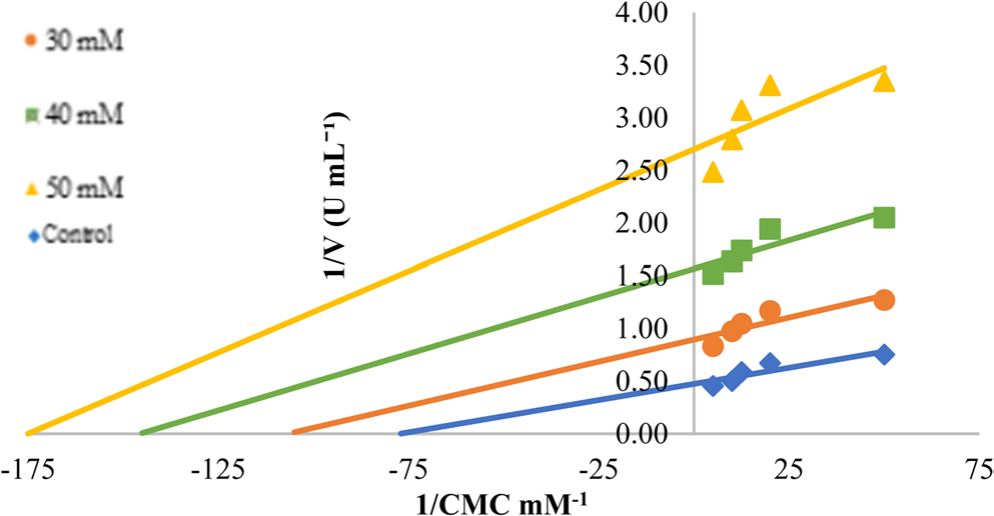
Inhibitory effect of MgSO_4_ on cellulase enzyme

**Fig. 20:**
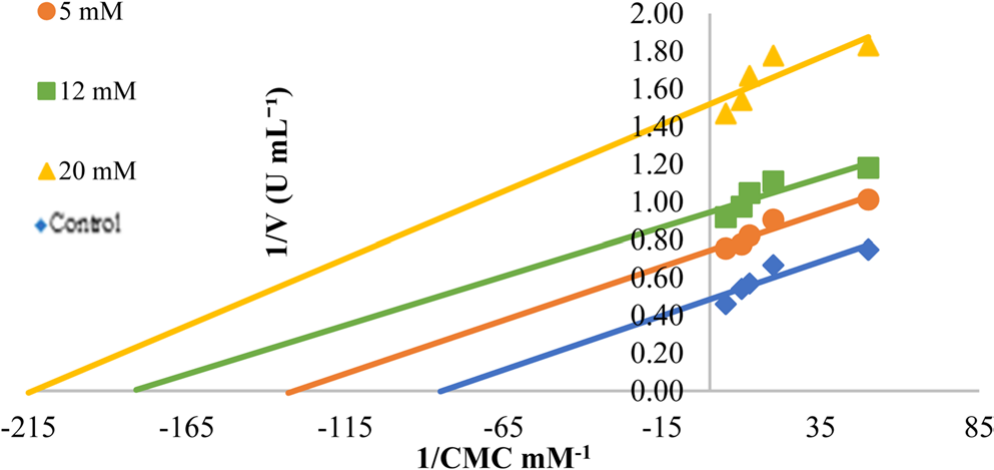
Inhibitory effect of KI on cellulase enzyme

**Fig. 21:**
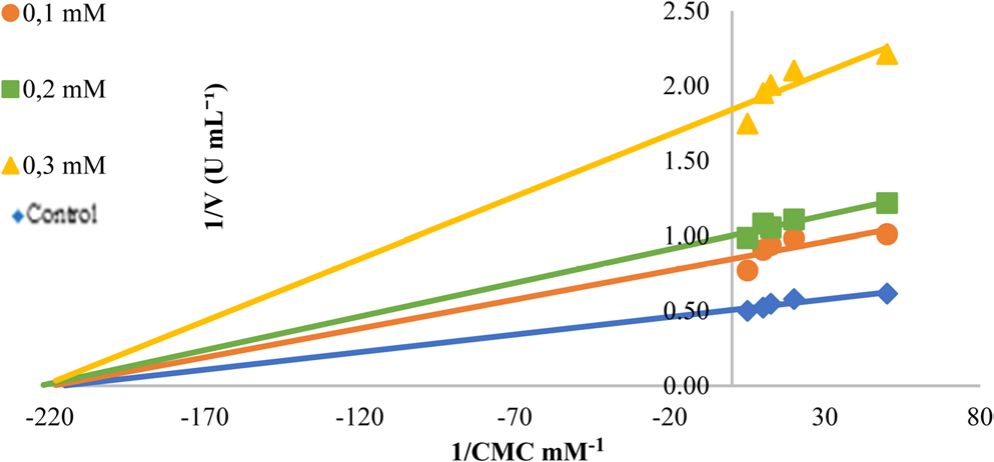
Inhibitory effect of SDS on cellulase enzyme

The inhibitory effects of several chemical compounds on cellulase enzyme activity were investigated, and the concentrations causing 50% inhibition (IC_50_ values) were determined. Five different concentrations of EDTA, KCl, MgSO_4_, KI, and SDS were tested, and % activity versus inhibitor concentration ([I]) graphs were plotted. According to the results, the IC_50_ values were found to be 25.67 mM for EDTA, 38.51 mM for KCl, 38.51 mM for MgSO_4_, 12.37 mM for KI, and 0.1791 mM for SDS. (*p* < 0.05, *n* = 3) (Table 4) (Figs. 12, 13, 14 and 15, 16, and 17) (Tables 5, 6, 7, 8, 9, 10, 11, 12, 13, 14, 15) (Fig. 18, 19, 20, and 21).

**Table 4:** IC_50_ Values of selected compounds affecting cellulase enzyme activity

Chemical Compound	IC_50_ (mM)
EDTA	25.67
KCl	38.51
MgSO_4_	38.51
KI	12.37
SDS	0.1791

**Table 5:** Effect of EDTA on cellulase

EDTA (mM)	Relative Activity (%) ± SD
0.00	100.00 ± 1.05^ab^
1.00	91.89 ± 0.06^a^
2.00	83.64 ± 6.13^ab^
5.00	78.61 ± 5.93^ab^
10.00	71.30 ± 4.99^bc^
20.00	59.54 ± 4.13^cd^
50.00	41.80 ± 6.75^de^
100.00	16.11 ± 3.16^e^

Values are expressed as mean ± standard deviation. Values followed by different letters within the same column are significantly different at *p* < 0.05 (*n* = 3)

**Table 6:** Effect of KCl on cellulose

KCl (mM)	Relative Activity (%) ± SD
0.00	100.00 ± 1.22^a^
10.00	84.42 ± 4.20^b^
20.00	70.13 ± 14.15^c^
40.00	45.45 ± 0.37^d^
60.00	33.77 ± 6.83^e^
80.00	25.97 ± 4.36^f^
100.00	15.58 ± 1.40^g^

Values are expressed as mean ± standard deviation. Values followed by different letters within the same column are significantly different at *p* < 0.05 (*n* = 3)

**Table 7:** Effect of MgSO_4_ on cellulase

MgSO_4_ (mM)	Relative Activity (%) ± SD
0.00	100.00 ±1.17^a^
5.00	91.94 ± 1.03^b^
10.00	85.57 ± 0.95^c^
20.00	65.07 ± 0.97^d^
50.00	39.27 ± 0.92^e^
100.00	16.04 ± 1.03^f^

Values are expressed as mean ± standard deviation. Values followed by different letters within the same column are significantly different at *p* < 0.05 (*n* = 3)

**Table 8:** Effect of KI on cellulase

KI (mM)	Relative Activity (%) ± SD
0.00	100.00 ±4.64^a^
1.00	82.45 ± 2.07^b^
5.00	61.77 ± 4.19^c^
10.00	48.94± 6.33^d^
20.00	31.57 ± 7.30^e^
30.00	20.99 ±2.31^f^

Values are expressed as mean ± standard deviation. Values followed by different letters within the same column are significantly different at *p* < 0.05 (*n* = 3)

**Table 9:** SDS effect of cellulase (mean ± SD)

SDS (mM)	Relative Activity (%) ± SD
0.00	100.00± 1.19^a^
0.01	95.04 ± 1.43^a^
0.03	86.86 ± 1.73^b^
0.05	75.58 ± 3.45^c^
0.10	66.45 ± 4.98^d^
0.30	32.12 ± 6.75^e^

Values are expressed as mean ± standard deviation. Values followed by different letters within the same column are significantly different at *p* < 0.05 (*n* = 3)

**Table 10:** İnhibition type of selected compounds affecting cellulase enzyme

Inhibitor	Preliminary Inhibition Type
EDTA	Uncompetitive
KCl	Non-competitive
MgSO_4_	Uncompetitive
KI	Uncompetitive
SDS	Non-competitive

**Table 11:** Inhibitory effect of EDTA on cellulase enzyme

CMC (mM)	EDTA/ 1/V			
	0	20	25	30
0.02	0.80 ± 0.02 ^aC^	1.33 ± 0.06 ^aB^	1.43 ± 0.03 ^aB^	2.01 ± 0.10 ^aA^
0.05	0.70 ± 0.01 ^bD^	1.27 ± 0.01 ^abC^	1.40 ± 0.04 ^aB^	2.00 ± 0.11 ^aA^
0.08	0.61 ± 0.02 ^cD^	1.22 ± 0.05 ^bcC^	1.33 ± 0.01 ^bB^	1.95^a^±0.05^bA^
0.1	0.56 ± 0.01 ^dD^	1.16 ± 0.02 ^cdC^	1.27 ± 0.03 ^bcB^	1.80 ± 0.09 ^bcA^
0.2	0.49 ± 0.01 ^eD^	1.10 ± 0.01 ^dC^	1.23 ± 0.01 ^cB^	1.70 ± 0.03 ^cA^

Values are expressed as mean ± standard deviation. Different letters indicate statistically significant differences according to Duncan’s multiple range test at *p* < 0.05 (lowercase letters denote column comparisons, while uppercase letters denote row comparisons; *n* = 3)

**Table 12:** Inhibitory effect of KCl on cellulase enzyme

CMC (mM)	KCL/ 1/V			
	0	30	40	50
0.02	0.63±0.00 ^aC^	0.74±0.01 ^aB^	0.84±0.11 ^aB^	1.05±0.02 ^aA^
0.05	0.50±0.01 ^bC^	0.70±0.00 ^bB^	0.71±0.02 ^bB^	0.86±0.04 ^bA^
0.08	0.49±0.04 ^bD^	0.60±0.01 ^cC^	0.66±0.01 ^bcB^	0.83±0.00 ^bcA^
0.1	0.46±0.02 ^bcD^	0.55±0.02 ^dC^	0.62±0.00 ^bcB^	0.80±0.00 ^cA^
0.2	0.44±0.00 ^cD^	0.51±0.00 ^eC^	0.59±0.01 ^cB^	0.73±0.02 ^dA^

Values are expressed as mean ± standard deviation. Different letters indicate statistically significant differences according to Duncan’s multiple range test at *p* < 0.05 (lowercase letters denote column comparisons, while uppercase letters denote row comparisons; *n* = 3)

**Table 13:** Inhibitory effect of MgSO_4_ on cellulase enzyme

CMC (mM)	MgSO_4_ 1/V			
	0	30	40	50
0.02	0.75±0.01 ^aD^	1.27±0.02 ^aC^	2.05±0.09 ^aB^	3.35±0.14 ^aA^
0.05	0.67±0.01 ^bD^	1.17±0.08 ^bC^	1.94±0.01 ^bB^	3.31±0.13 ^aA^
0.08	0.58±0.00 ^cD^	1.04±0.01 ^cC^	1.74±0.06 ^cB^	3.07±0.12 ^bA^
0.1	0.50±0.00 ^dD^	0.97±0.01 ^cC^	1.63±0.03 ^cB^	2.79±0.08 ^cA^
0.2	0.45±0.01 ^eD^	0.83±0.06 ^dC^	1.51±0.03 ^dB^	2.48±0.08 ^dA^

Values are expressed as mean ± standard deviation. Different letters indicate statistically significant differences according to Duncan’s multiple range test at *p* < 0.05 (lowercase letters denote column comparisons, while uppercase letters denote row comparisons; *n* = 3)

**Table 14:** Inhibitory effect of KI on cellulase enzyme

CMC (mM)	KI 1/V			
	0	5	12	20
0.02	0.74±0.02 ^aD^	1.01±0.01^aB^	1.18±0.02 ^aA^	0.91±0,04 ^aC^
0.05	0.66±0.00 ^bD^	0.90±0.02 ^bC^	1.10±0.00 ^abB^	1.77±0,05 ^bA^
0.08	0.57±0.00 ^cD^	0.82±0.09 ^cC^	1.05±0.01 ^bcB^	1.67±0,04 ^cA^
0.1	0.54±0.00 ^dD^	0.77±0.00 ^dC^	0.97±0.12 ^cdB^	1.54±0,01 ^dA^
0.2	0.46±0.01 ^eD^	0.75±0.05 ^eC^	0.91±0.01 ^dB^	1.47±0,00 ^eA^

Values are expressed as mean ± standard deviation. Different letters indicate statistically significant differences according to Duncan’s multiple range test at *p* < 0.05 (lowercase letters denote column comparisons, while uppercase letters denote row comparisons; *n* = 3)

**Table 15:** Inhibitory Effect of SDS on Cellulase Enzyme

CMC (mM)	SDS 1/V			
	0	0.1	0.2	0.3
0.02	0.62±0.02 ^eA^	1.01±0.03 ^bB^	1.22±0,03 ^dC^	2.21±0.02 ^cD^
0.05	0.58±0.02 ^dA^	0.98±0.00 ^bB^	1.11±0,00 ^cC^	2.10±0.07 ^bcD^
0.08	0.55±0.03 ^cA^	0.94±0.01 ^abB^	1.01±0,00 ^bC^	2.00±0.03 ^bcD^
0.1	0.52±0.00 ^bA^	0.91±0.27 ^abB^	1.08±0,00 ^bcB^	1.96±0.06 ^bC^
0.2	0.50±0.04 ^aA^	0.77±0.00 ^aB^	0.99^aC^±0.02	1.75±0.03 ^aD^

Values are expressed as mean ± standard deviation. Different letters indicate statistically significant differences according to Duncan’s multiple range test at *p* < 0.05 (lowercase letters denote column comparisons, while uppercase letters denote row comparisons; *n* = 3)

## Discussion and recommendations

Although the chemical industry has provided substantial benefits to modern society, increasing environmental and health concerns associated with conventional production processes have accelerated the shift toward biotechnological approaches. This transition has highlighted the importance of stress-tolerant and industrially relevant microorganisms and the functional potential of their biomolecules for industrial applications. Advances in enzyme-based technologies, together with their cost-effectiveness and environmental sustainability, have significantly strengthened the role of biotechnology in recent years (Güven 2011). In this context, microbial cellulases have gained particular attention due to their central role in the sustainable conversion of lignocellulosic biomass into value-added products and their relevance to both fundamental research and industrial process optimization (Ilić et al. 2023).

The characteristics exhibited by the cellulase enzyme obtained from the *B. licheniformis* isolate demonstrate a potential alignment with the industrial expectations mentioned in the introduction. The isolation of the strain from a thermal environment (hot spring) suggests that it may offer an advantage in terms of enzyme stability during certain stages of food processing that require elevated temperatures. Specifically, the cellulase index of 5.14, which is noteworthy when compared with previous literature, provides positive data regarding the effectiveness of this strain in degrading cellulosic structures. This indicates the possibility of contributing to efficiency in processes such as fruit juice clarification, where the degradation of plant cell walls is a primary objective.

Furthermore, the preservation of enzymatic activity even in a partially purified state suggests that this cellulase could be considered a cost-effective option for applications where high-purity enzymes are not strictly mandatory. For instance, the potential use of such isolates could be evaluated in areas such as balancing viscosity in brewing or supporting dough structure in bakery products. Despite the fact that incubation conditions were not fully optimized, these results provide an insight into the strain’s robustness under the tested conditions. These findings suggest that the investigated isolate warrants further evaluation as a potential biocatalyst candidate for enhancing efficiency in the food sector.

In the literature, cellulolytic index values reported for bacterial isolates range between 1.57 and 8 (Islam et al. 2021), while some studies have reported comparatively lower values. For instance, a study presented at the International Conference on Green Agro-industry and Bioeconomy reported a maximum enzyme index of 2.32 for bacterial cellulase activity (Wijayanti et al. 2020); In addition, cellulases derived from various arthropod sources have shown average index values between 3.51 and 5.96 (Gupta et al. 2012). Furthermore, cellulase index values have been reported to vary widely, ranging from 0.42 to 4.0 (Hatami et al. 2008) and from 4.24 to 10.36 (Lu et al. 2005), highlighting the strong influence of organism-specific characteristics and experimental parameters on cellulolytic activity. When evaluated collectively, these studies demonstrate that cellulolytic index values exhibit substantial variability depending on the microorganism, strain, and experimental conditions. In this context, the cellulolytic index value of 5.14 obtained in the present study falls within the range reported in the literature and indicates that the investigated isolate possesses a moderate to high cellulolytic potential. However, previous studies have shown that variations in incubation time, temperature, and medium composition can significantly enhance cellulolytic index values. In the present study, systematic evaluation of different incubation conditions could not be performed due to limitations in available laboratory facilities, which represents one of the limitations of this work. Therefore, future studies focusing on the optimization of incubation parameters may contribute to achieving higher cellulolytic index values. The OE-1 strain demonstrated strong cellulolytic potential when evaluated on CMC-LB agar, as evidenced by the previously reported 29 mm hydrolysis zone and 5.14 enzyme index (Eren and Sakiroğlu, in press). Although these values were not newly generated in the present study, they were referenced to contextualize the strain’s enzymatic capacity relative to existing literature. Notably, literature data indicate that such values are comparatively higher than those reported for many wild-type cellulolytic bacteria, highlighting cellulolytic potential of the OE-1 strain. These findings support the suitability of OE-1 as a promising candidate for further biotechnological applications involving cellulase-mediated biomass conversion.

In the present study, purification of the crude cellulase was attempted using ammonium sulfate fractionation from 10% to 80% saturation. Despite repeated attempts, no enzymatic activity was recovered in the resulting fractions. This outcome is likely a consequence of low initial cellulase secretion levels under the applied non-optimized incubation conditions. Consistent with this, protein quantification revealed very low protein concentrations in the crude supernatant. Accordingly, the weak or absent protein bands observed in SDS-PAGE analysis likely reflect low protein recovery rather than a limitation of the purification methodology itself.

Alkaline-active cellulases have garnered considerable interest in industrial and biotechnological applications due to their ability to function efficiently under high pH conditions, which are characteristic of numerous commercial processes. Such enzymes are particularly valuable in the detergent industry, where alkaline cellulases enhance stain removal and fabric care in high-pH formulations, demonstrating compatibility with surfactants and thermal stability (Hill et al. 2006). Moreover, alkaline tolerant cellulases play a pivotal role in the biofuel industry, facilitating the saccharification of lignocellulosic biomass into fermentable sugars in pre-treatment steps that frequently occur at elevated pH levels (Hill et al. 2006; Hmad & Gargouri, 2017). Beyond these sectors, alkaline cellulases are applied in pulp and paper processing, improving fiber properties and reducing energy consumption, and are being explored for waste management and sustainable biomass valorisation (Kuhad et al. 2011; Maravi and Kumar 2021). Collectively, these applications underline the strategic importance of alkaline-functioning cellulases in advancing sustainable and efficient industrial bioprocesses.

Several studies have reported cellulase enzymes with optimal activity at alkaline pH values. For instance, a thermostable alkaline cellulase from *Bacillus* sp. KSM-S237 exhibited maximum activity at pH 8.6–9.0 (Hakamada et al. 1997). Similarly, alkaline cellulase from *Bacillus pumilus* VLC7 showed optimum activity at pH 9.0 and retained substantial activity across a broad pH range (Yilmaz and Gurkok 2025) Additionally, a *Bacillus subtilis*-derived CMC displayed optimal activity at pH 9.2 (Deka et al. 2013). Furthermore, several independent studies have reported the presence of enzymes exhibiting optimum activity at elevated pH levels, highlighting the occurrence of alkaline-active cellulolytic enzymes in diverse microbial sources (Vega et al. 2012; Arya et al. 2025). previous studies have demonstrated the existence of cellulolytic enzymes with optimum activity under alkaline conditions. In agreement with these reports, the cellulase characterized in the present study exhibited maximum activity at pH 9.0, supporting its classification as an alkaline-active enzyme.

The observed changes in enzyme activity across different pH values are fundamentally linked to the electrostatic conditions within the cellulase active site. The catalytic mechanism of glycoside hydrolases, including cellulases, critically relies on specific acidic amino acid residues, primarily aspartate and glutamate, which act as proton donors and nucleophiles. The ionization state of the carboxyl groups on these key residues is highly pH dependent the supernatant served as the enzyme source. Alterations in the environmental pH can disrupt the optimal protonation states required for proper substrate binding and catalysis, thereby reducing overall enzyme activity. In this context, the ability of the enzyme to retain activity under alkaline pH conditions, together with the observation of a relatively promising cellulolytic index despite a limited total enzyme yield, suggests that the investigated bacterial isolate may possess potentially noteworthy catalytic properties under specific conditions. These features indicate that, particularly for biotechnological and industrial processes requiring cellulases active at alkaline pH, the isolate may warrant further investigation through targeted optimization studies, without implying direct industrial applicability at the present stage.

Previous studies have reported the use of glycine-NaOH buffers for assaying crude cellulase activity. In one study, crude cellulase was incubated in 50 mM glycine-NaOH buffer (Deka et al. 2011). while another investigation supplemented a crude cellulase solution with the same buffer concentration (Zainuddin et al. 2024). Similarly, in research conducted on supernatants obtained from different sites of Van Lake, Turkey, as a source of crude enzyme, 50 mM glycine-NaOH buffer was employed to assess enzymatic activity (Yilmaz and Gurkok 2025). On the other hand, partially purified cellulase from Bacillus spp. was assayed using 100 mM glycine-NaOH buffer (Shikata et al. 1990). The crude cellulase investigated in this study demonstrated substantial activity under alkaline conditions, consistent with previous reports on alkaline cellulases. Alkaline cellulases have been routinely assayed under glycine-NaOH buffer systems at pH 8–10, showing significant catalytic activity in high-pH environments, which is suitable for evaluating cellulase performance (Arya et al. 2025). Similarly, thermo-alkalistable cellulases from Geobacillus sp. have been characterized using 100 mM glycine-NaOH buffer at pH 8–9, further supporting the use of this buffer system for activity assessment under alkaline conditions (Deka et al. 2013).

In the present study, the crude cellulase demonstrated maximum activity under 100 mM glycine-NaOH buffer at alkaline pH, aligning with the previous observations and indicating that the enzyme is tolerant to both elevated buffer concentration and alkaline conditions. The observed tolerance of the enzyme to both elevated pH and buffer concentration indicates a degree of robustness, which may be advantageous for industrial applications; however, further studies are required to evaluate its stability and performance under process-relevant conditions.

It is well established that, in addition to pH, buffer composition can significantly influence enzyme activity by modulating the microenvironment of the active site and the ionization state of catalytic residues (Forero et al. 2023). The observation that the crude cellulase exhibited maximum activity in 100 mM glycine-NaOH buffer suggests that the enzyme is well adapted to alkaline conditions and maintains catalytic efficiency under relatively high buffering capacity. The tolerance of the enzyme to both alkaline pH and elevated buffer concentration may therefore indicate a degree of robustness, although further studies are required to evaluate its stability and performance under process-relevant conditions.

A study conducted in the Himalayan region reported that cellulase derived from *Bacillus licheniformis* exhibits optimal activity at 60 °C (Shyaula et al. 2023). Bischoff et al. (2006) isolated and purified cellulase from *B. licheniformis* B-41,361, reporting an optimal activity of 65 °C and a thermal stability of 60 °C. In a separate study, Seo et al. (2013) evaluated the thermal properties of the cellulase complex subunits, determining an optimal temperature of 70 °C for endoglucanase and 50 °C for β-glucosidase. Additional studies have reported that the optimal temperatures of various *B. licheniformis* cellulases range between 40, 65, and 70 °C (Azadian et al. 2016; Kazeem et al. 2016; da Silva et al. 2021).

In the present study, the crude cellulase exhibited maximum activity at 60 °C, indicating a promising for industrial applications. Its ability to retain activity at elevated temperatures enhances suitability for thermally demanding processes while simultaneously minimizing the risk of microbial contamination. These characteristics underscore the enzyme’s applicability in both biotechnological and industrial contexts. Variations in reported optimal temperatures across studies can be attributed to several factors. Differences in enzyme preparation and purification from crude extracts to fully purified forms can significantly affect measured activity, as crude enzymes may confer greater resistance to environmental conditions while potentially reducing catalytic performance. Moreover, several studies have reported that the subunits of cellulase exhibit distinct optimal temperatures. Cellulase is a multimeric enzyme composed of three subunits, and consequently, more purified or complexed enzyme forms may display maximum activity at different temperatures. In addition, the source of the enzyme is a critical factor. Compared to cellulase derived from a thermophilic bacterium in our study, *B. licheniformis* cellulases isolated from goat rumen have been reported to exhibit optimal activity over a wide range of 35–70 °C (Seo et al. 2013; Rabapane et al. 2025).

Studies on the storage stability of cellulases have indicated that immobilized enzymes generally retain higher activity under long-term cold storage. For instance, Li et al. (2019) reported that an immobilized cellulase retained 71.2% of its activity after one month at 4 °C, while the free enzyme showed a comparable retention (72.7%) after only 7 days. Similarly, John et al. (2023) observed 89% retention for an immobilized enzyme after one month. Other report show varying results, with activities ranging from 44–49 days (Hojnik Podrepšek et al. 2022).

In the present study, the crude extract without purification or immobilization retained approximately 70% of its initial activity after 8 days at 4 °C, with no detectable loss during the first 100 h. While industrial enzymes often require deep-freezing (-20 °C or -80 °C) for long-term storage, the observed moderate stability under refrigeration conditions may offer certain practical considerations for short-term handling. These findings suggest that with further purification or appropriate immobilization strategies, the enzyme might potentially achieve stability levels comparable to those reported for existing immobilized systems.

The kinetic parameters of the cellulase enzyme characterized in this study, with a K_m_ of 0.1691 mg/mL and a V_max_ of 2.29 U.mL^− 1^, indicate favorable substrate affinity and catalytic efficiency. Compared to literature-reported values, these parameters appear favorable: cellulase from *Bacillus licheniformis* has previously been reported to have a Vmax of 0.493 IU/mL, while other studies have demonstrated Vmax values of 284 µM/min. Subunits such as β-1,4-endoglucanase, β-1,4-exoglucanase, and β-glucosidase have shown Vmax values of 2.5 IU/mL, 0.8 IU/mL, and 0.084 IU/mL, respectivel (Amore et al. 2013; Pandey et al. 2014; da Silva et al. 2021). Furthermore, kinetic parameters reported for other *Bacillus licheniformis* or *Bacillus subtilis* cellulases vary: for example, the PANG L cellulase exhibited a K_m_ of 1.8 mg/mL and a V_max_ of 10.92 µg/mL/min (Shyaula et al. 2023), CD001 cellulase showed a K_m_ of 0.996 mM and a V_max_ of 1.647 U/mL (Malik and Javed 2021) and AS3 cellulase reported a K_m_ of 0.13 mg/mL and a V_max_ of 3.38 U/mg (Deka et al. 2013). Relative to these reports, the crude enzyme extract in this study demonstrates a significantly lower K_m_, suggesting higher substrate affinity, while its V_max_ is comparable to both purified enzymes and individual subunits. These findings highlight that, even without purification or immobilization, the crude enzyme extract exhibits favorable kinetic properties that may be advantageous for industrial applications.

In the present study, the activation energy (E_a_) for the cellulase was found to be 2.82 kcal/mol, which is notably lower than many reported values for microbial cellulases. This low Ea, along with an activation enthalpy (∆H) of 2.16 kcal/mol, indicates that the enzyme significantly lowers the energy barrier for the hydrolysis reaction, facilitating a more efficient transition state. From a thermophysical perspective, these low values suggest that the catalytic active site likely governed by acidic residues such as aspartate and glutamate provides an optimal electrostatic environment that stabilizes the enzyme-substrate complex with minimal thermal energy input. Although the calculated Ea indicates efficient catalytic potential, this value should be interpreted with caution as the assays were performed using a crude enzyme preparation, where the presence of other proteins or metabolites might influence the apparent kinetics. Furthermore, the low Q_10_ value (1.22) observed in this study reflects a relative independence of the reaction rate from sudden temperature fluctuations, reinforcing the idea of a stable and robust catalytic framework. Notably, recent studies have also reported low activation energy values for Bacillus cellulases, attributing this to a stronger enzyme-substrate complex and reduced energy barriers for the reaction (Tahir et al. 2025).

In the SDS-PAGE analysis, the observation of multiple faint bands instead of a single intense band suggests that the Sephadex G-100 chromatography achieved only partial purification. The presence of several bands indicates that contaminating proteins were not entirely removed, which explains the observed decline in Specific Activity. While the molecular weights for *B. licheniformis* cellulases are typically reported between 54.5 and 65.8 kDa (De Araújo et al. 2019; Elsababty et al. 2022), the low intensity of the bands in our study reflects the low initial enzyme expression of the isolate. The fact that multiple bands were visible, albeit faintly, confirms that the protein was not degraded but was present in a multi-protein mixture at low concentration. This technical limitation could be addressed in future studies by employing more selective techniques such as affinity chromatography or ion-exchange chromatography.

In the literature, studies focusing on the purification of cellulase from *Bacillus licheniformis* report that, following classical purification steps such as ammonium sulphate precipitation and dialysis, the purification coefficient decreases to approximately 0.3, the purification yield remains within the range of 38–45%, and, concomitantly, reductions in both enzyme activity and specific activity are observed when these measurements are performed on crude cellulase extracts (Wijayanti et al. 2020). Similar trends have been reported in the literature for cellulase purification from *B.licheniformis*. For instance, partial purification of cellulase from strain Z9 resulted in a dramatic reduction in total protein content from 157.5 mg to 2.3 mg and an overall yield of 3.07% following ammonium sulphate precipitation and gel filtration chromatography (Elsababty et al. 2022). Another study on a different *B. licheniformis* isolate reported enzyme recovery of 38% after simple precipitation steps, highlighting the challenge of maintaining high enzyme yield during purification (Sharma et al. 2025). In the present study, similar trends were observed: the enzyme yield decreased to 8.54%, the purification coefficient dropped to 71%, and both enzyme activity and specific activity declined. These findings are consistent with the literature, indicating that protein loss and reduced enzymatic yield are common outcomes during classical purification of *B. licheniformis* cellulases. The similar results obtained in the present study likely reflect the technical challenges associated with processing biological material with a low initial enzyme concentration, stemming from the cellulase production capacity of *B. licheniformis* strains. In strains with limited cellulase expression levels, the occurrence of protein losses during the successive stages of the purification process is an expected outcome (Lee 2017). In this context, the reduction in total protein content observed after chromatography reflects not a limitation in the effectiveness of the applied purification strategy, but rather the natural consequence of processing biological material with a low initial enzyme concentration. Although the application of alternative or additional purification steps is theoretically possible, the approach adopted in this study was determined in accordance with the available laboratory infrastructure and technical capabilities. Due to the limited accessibility of advanced chromatographic systems, affinity columns, or equipment for recombinant protein purification, ammonium sulphate precipitation and classical chromatographic methods, which are widely used in the literature and readily applicable under basic laboratory conditions, were preferred. Nevertheless, the purification yields and enzyme activity obtained are consistent with values reported for comparable biological systems, indicating that the applied approach provided results consistent with the intended objectives within the scope of this work.

In the present study, the observation of a notable enzyme index despite a low total enzyme yield suggests that the enzyme may possess favorable catalytic properties, even though its expression levels are relatively low under the tested conditions. Accordingly, systematic optimization of production parameters might enhance the recovery of enzyme yields. These findings indicate that the investigated bacterial isolate warrants further evaluation as a potential candidate for biotechnological applications involving cellulase production. Although some researchers have reported a linear correlation between cellulolytic enzyme index values and the logarithmic enzyme quantity (Teather and Wood 1982), several other studies have demonstrated that a high enzyme index does not necessarily correlate linearly with enzyme yield or total enzymatic activity, even when evaluated at larger production volumes (Sadhu and Maiti 2013; Liang et al. 2014). In those studies, this discrepancy has been attributed either to enzyme production levels in liquid culture being below the detection threshold or to the inherently low enzyme production capacity of the bacterial strain under the tested conditions (Liang et al. 2014). Similarly, the limitation encountered in the present study may be considered one of the constraints of the experimental design. Addressing this limitation would likely require systematic optimization of incubation parameters, including temperature, incubation time, and medium composition. Furthermore, analysis of the expression levels of genes associated with cellulase production could provide direct evidence as to whether the enzyme is indeed produced at low levels; however, such analyses could not be performed due to limitations in laboratory infrastructure and technical resources. Nevertheless, these approaches are expected to guide future studies and facilitate a more comprehensive evaluation of the cellulase production potential of the bacterial isolate. Taken together, our results indicate that the observed discrepancy between cellulolytic index and enzyme yield reflects intrinsic biological and methodological constraints rather than experimental inadequacy and underscores the need for targeted optimization strategies.

EDTA has been widely recognized as a metal-chelating agent that modulates cellulase activity by sequestering essential metal ions. Previous studies have reported that 5 mM EDTA reduced relative cellulase activity to 33% (Malik and Javed 2024) and 21% (Malik and Javed 2021) for purified enzymes. Furthermore, 1 mM EDTA was shown to variably influence the same enzyme’s activity, ranging from 12% to 89%, depending on the reaction environment (Okonkwo 2019), highlighting the sensitivity of enzyme inhibition to assay conditions. Mechanistically, EDTA may exert its inhibitory effect either by removing metal ions from the enzyme–substrate complex (Ali et al. 2022) or by displacing metal cofactors within the enzyme structure itself, thereby inducing inactivation (Schmid 1997). In the present study, the higher EC₅₀ value observed for EDTA (25.67 mM) is likely attributable to the use of crude enzyme extract, in which endogenous metal ions may partially buffer the chelating effect of EDTA. Additionally, the uncompetitive inhibition pattern observed in our kinetic analysis supports the notion that EDTA predominantly targets the enzyme-substrate complex for metal ion removal, rather than the free enzyme, thereby modulating catalytic efficiency in a context-dependent manner. EDTA has generally been reported as an inhibitor of cellulase activity due to its metal-chelating properties; however, the literature indicates that its effect is highly dependent on binding context and experimental conditions. Previous studies on purified endoglucanases have shown that low EDTA concentrations (e.g., 5 mM) cause a decrease in ellipticity at 217 nm and a pronounced increase in the apparent melting temperature (T_m_), resulting in enhanced structural stability and increased catalytic turnover (kcat). (Naika and Tiku 2011). In contrast, in the present study, the enzyme was evaluated in a crude extract and exposed to higher EDTA concentrations. Under these conditions, EDTA is likely to chelate not only loosely bound or inhibitory metal ions but also metal ions essential for catalytic activity. This behavior is consistent with the observed uncompetitive inhibition pattern, suggesting preferential interaction with the enzyme–substrate complex and subsequent removal of associated metal ions. Therefore, the divergent effects of EDTA reported in the literature can be consistently explained by differences in enzyme purity, the type and availability of metal ions in the reaction environment, and EDTA concentration.

Proteomic analyses using native SDS-PAGE have demonstrated that conventional SDS-PAGE running buffers containing 0.1% SDS and 1 mM EDTA can mobilize a substantial fraction of protein-bound metal ions, such as Zn²⁺, from metalloproteins. In one study, approximately 74% of Zn²⁺ was released under these conditions, indicating that SDS, particularly in combination with chelating agents, can disrupt metal-binding sites during electrophoretic separation (Nowakowski et al. 2014). Proteins such as cellulases, which possess multiple positively charged residues, are generally more sensitive to metal ions than to protons. As a negatively charged anionic detergent, SDS can interact strongly with these positively charged regions, and even low concentrations of SDS are sufficient to induce enzyme denaturation. Consistently, it has been reported that exposure to 1% SDS reduced cellulase activity to as low as 15% (Doan et al. 2024). Mechanistically, SDS may contribute to enzyme inactivation by binding metal ions that serve as cofactors, thereby destabilizing the structural integrity of the enzyme. Consequently, the combined effects of electrostatic interactions between negatively charged SDS molecules and the protein, together with disruption of metal cofactor associations, can lead to pronounced inhibition of cellulase activity.

Our findings are consistent with previous studies reporting strong inhibitory effects of SDS on cellulase activity. For instance, 1 mM SDS reduced enzymatic activity to 26.8%, while 5 mM nearly completely inhibited cellulase, an effect attributed to the disruption of non-covalent interactions (Ouyang et al. 2025). Similarly, in another study, addition of 0.1% SDS relative to the enzyme-substrate concentration decreased cellulase activity to 55%, further corroborating the sensitivity of cellulase to SDS-mediated denaturation (Devi et al. 2022). These observations align with the low EC₅₀ value determined in the present study (0.1791 mM), underscoring that even relatively small SDS concentrations can drastically impair catalytic efficiency, likely through combined disruption of electrostatic interactions and metal cofactor binding essential for structural integrity.

When compared with EDTA (EDTA EC₅₀ 25.67 mM), SDS exhibited a markedly stronger inhibitory effect on cellulase activity, as evidenced by its substantially lower EC₅₀ value (0.1791 mM). This pronounced difference suggests that SDS primarily inhibits the enzyme through direct denaturation and disruption of electrostatic and metal cofactor interactions, whereas EDTA exerts a more gradual, context-dependent inhibitory effect mainly via metal ion chelation.

The literature reports variable and sometimes contradictory effects of both MgSO₄ and KCl on cellulase activity. The literature reports that the effects of MgSO₄ and Mg²⁺ ions on cellulase activity are highly variable and depend on the enzyme source, ionic form, and concentration. In a study using cellulase derived from *B.licheniformis*, 5 mM MgSO₄ was reported to inhibit enzyme activity by only approximately 7% (Azadian et al. 2016). In contrast, another study on fungal-derived cellulase indicated that even a low concentration of 1 mM MgSO₄ reduced enzyme activity by approximately 70% when CMC was used as the substrate (Okonkwo 2019). Similarly, Mg²⁺ ions at a concentration of 10 mM were reported to inhibit cellulase activity by about 30% (Wang et al. 2012). Conversely, in *Mucor circinelloides* derived cellulase, 5 mM MgCl₂ increased enzyme activity by approximately 27% (Saha 2004).

Taken together, these results suggest that the effect of magnesium on cellulase cannot be explained by a universal inhibitory mechanism; rather, it may act as an activator or inhibitor depending on the enzyme source, substrate, and chemical form of magnesium. Notably, cellulase derived from *Bacillus licheniformis* appears to be relatively resistant to low magnesium concentrations, and the IC₅₀ value of 38.51 mM determined in the present study indicates that this enzyme exhibits only weak inhibition in the presence of MgSO₄. This finding suggests that Bacillus-derived cellulases can be used in industrial processes containing MgSO₄ or other magnesium salts in a stable and reliable manner.

Similarly, the effects of KCl on cellulase activity are highly variable. Some studies reported that 2–3 M KCl enhanced cellulase activity (Hua et al. 2015), and in halophilic and alkaliphilic *Bacillus* strains, 3 M KCl increased activity by approximately tenfold (Zhang et al. 2012). In contrast, 4 M KCl reduced β-1,4-endoglucanase activity by nearly 48% (Liang et al. 2011). While some studies reported stimulatory effects of KCl on endoglucanase activity (Hirasawa et al. 2006), low concentrations such as 5 mM KCl had no significant effect (Trivedi et al. 2011). In our study, the IC₅₀ of KCl was determined as 38.51 mM, indicating that its effect on cellulase is dependent on concentration, enzyme source, and experimental conditions.

This comparative evaluation demonstrates that the effects of Mg²⁺ and K⁺ salts on cellulase activity can be markedly different and sometimes opposite. The limited and inconsistent literature highlights the importance of quantitative investigations in this field, and our study provides a valuable contribution by determining the IC₅₀ values of MgSO₄ and KCl on cellulase activity.

Potassium iodide (KI) has not been specifically evaluated for its effect on cellulase activity in the current literature; most studies focus on classical metal ions (e.g., Mg²⁺, Ca²⁺, K⁺, Zn²⁺) and their influence on cellulolytic enzymes. This highlights a significant gap in the literature regarding iodide ions (I⁻) and their potential modulatory effects on cellulase kinetics. In the present study, we determined that the IC₅₀ value of KI is 12.37 mM, providing the first quantitative evidence of its effect on cellulase activity. This finding not only fills a notable gap in the literature but also suggests that KI can exert a measurable inhibitory effect on cellulase, which may have implications for enzymatic processes in which iodide is present.

Taken together, these results indicate that Mg²⁺, K⁺, and I⁻ salts can act as modulators of cellulase activity, with their effects varying depending on concentration and enzyme source. Notably, *Bacillus licheniformis*-derived cellulase appears relatively resistant to MgSO₄ and KCl, while KI exerts a stronger inhibitory effect at lower concentrations. These findings not only fill important gaps in the literature but also suggest that Bacillus cellulases can be used reliably in industrial processes involving magnesium- or potassium-containing environments, whereas the presence of iodide may require careful consideration due to its inhibitory potential.

The inhibition types of the tested compounds were preliminarily determined as follows: EDTA, MgSO₄, and KI exhibited uncompetitive inhibition, while KCl and SDS showed non-competitive inhibition. It should be noted that these classifications are preliminary, as Lineweaver-Burk plots are sensitive to experimental variability. Uncompetitive inhibition indicates that the inhibitor binds only to the enzyme-substrate complex, reducing enzymatic activity, whereas non-competitive inhibition reflects inhibitor binding to an allosteric site, affecting enzyme activity regardless of substrate concentration. EDTA is a classical metal-chelating agent that can bind essential metal ions in metalloenzymes, thereby altering enzyme conformation. Such interactions typically result in non-competitive or mixed inhibition; however, in our study, since cellulase was present in a crude extract, the effect of EDTA may have been limited or altered. This suggests that the inhibitory effect could have been observed more clearly if the enzyme had been purified, representing a limitation of this work.

Although the inhibition type of Mg²⁺ ions has not been directly reported in the literature, kinetic analyses suggest a competitive-like behavior (Wang et al. 2012). In our study, MgSO₄ exhibited a relatively high IC₅₀, indicating that *B.licheniformis* cellulase is relatively tolerant to Mg²⁺ and could maintain stability under industrial conditions.

Regarding KCl, the literature indicates that its inhibition type can vary depending on the source of cellulase and the substrate used, showing competitive or uncompetitive effects in different systems (Gundupalli et al., 2021). Our findings confirm that KCl exerts a moderate inhibitory effect based on IC₅₀ values, and that the inhibition mechanism may depend on the enzyme–substrate combination.

SDS reduces cellulase activity by blocking access to the active site and altering enzyme conformation. Although this effect is not directly classified under classical inhibition types, it can be interpreted as consistent with non-competitive or mixed inhibition behavior (Kjølbye et al. 2019). This finding highlights that surfactants can affect enzymes not only by obstructing the active site but also by altering molecular structure.

Overall, these results demonstrate that the inhibitory effects of different salts and surfactants on *B.licheniformis* cellulase are highly dependent on enzyme source, substrate, and chemical environment. Furthermore, IC₅₀ values provide quantitative insight into the relative potency of these inhibitors and help interpret the limitations associated with using crude enzyme extracts. This emphasizes the enzyme’s stability and optimization potential for industrial applications under varying ionic and surfactant conditions.

These findings indicate that the responses of cellulase to different ions and inhibitors are complex and specific, with the inhibition types and degrees of effect being directly related to the enzyme source and the chemical form used. Furthermore, they provide new insights into the effects of iodide ions, which have not been extensively studied in the literature, thereby filling an important knowledge gap in this area. The results suggest that *Bacillus licheniformis* cellulase can be used in a stable and reliable manner under various industrial conditions, particularly in environments containing magnesium and potassium, while careful optimization is required in systems containing iodide.

## Conclusion

In conclusion, the cellulase enzyme secreted by the *Bacillus licheniformis* OE-1 strain isolated from Diyadin thermal springs exhibits promising biochemical characteristics, notably its alkaline pH preference, thermostability, and apparent catalytic efficiency. While its production levels under current laboratory conditions remain modest, the enzyme’s favorable kinetic and stability parameters suggest a significant potential for future biotechnological applications. Further studies focusing on optimization of culture conditions and scale-up strategies are necessary to fully evaluate and harness the industrial value of this enzyme.

## Data Availability

The datasets generated and/or analyzed during the current study are available from the corresponding author upon reasonable request.
